# Hospital and Institutional News

**Published:** 1919-07-26

**Authors:** 


### HOSPITAL AND INSTITUTIONAL NEWS.

### WHO SHOULD SIGN APPEALS?

We think that the committee of the Hampstead General Hospital, and its secretary, Mr. Harold Wigg, are to be congratulated on having issued a recent letter to the press over the signature of local residents. The figurehead has ceased to be convincing. The local resident has no excuse if he is not sincere. The four arts of music, history, drama, and acting make a very agreeable quartet, and when we ask what purpose is common to Edward Elgar, John Fortescue, John Galsworthy, and Gerald du Maurier, it is pleasant to know that it is the welfare of their local hospital. They have indeed cause for concern, for not only is the hospital's account overdrawn by £10,000, but £10,000 is the limit of its borrowing powers as fixed by the Charity Commissioners. The writers remark that Hampstead is not only one of the prettiest, but one of the richest districts in London. It has only one general hospital. If the letter referred to does not produce a ready response there must be very little *esprit de corps* among Hampstead residents, who, by the way, include at least one millionaire to whom £10,000 is a trifle. At all events, the statement is admirably made, and the mode of its signature a welcome example of directness and good sense; and how rare these are in most appeals!

### THE "APPEAL AND PUBLICITY" DEPARTMENT OF A HOSPITAL.

On July 10, the advertisement columns of the *Times* contained a paragraph announcing a vacancy for an "Assistant Secretary for the Appeal and Publicity Department of a large London General Hospital." The salary offered is £250, and applicants "must have had experience in conducting campaigns for the raising of funds and possess initiative." An agency address was given, where further inquiry would doubtless reveal the identity of the institution concerned. The Hospital has long and earnestly impressed upon the voluntary hospital managers the importance of system and method in prosecuting the inevitable appeals for funds, and to a considerable extent this proposed appointment has our approval and sympathy. Presumably the advertisement means that the "assistant secretary" required is to work under the general secretary, and to devote his whole energies to the furtherance of appeals and publicity. We deem it very important that this should be so; the general secretary should unquestionably be cognisant of and responsible for all the acts of the official in charge of the sub-department in question. The salary offered is not, as such matters stand nowadays, a high one, but in the office of a hospital large enough to require the services of a specialist of this sort, there must necessarily be several avenues of promotion for a smart man; and the appointment now vacant should attract ample competition among the smarter and younger men of the clerical staffs of big hospitals.

### A SUCCESSFUL AMALGAMATION.

The Liverpool Country Hospital for Children at Heswall is remarkable in more ways than one, chiefly perhaps in allowing its patients an average stay of 243 days. Mr. Alfred Holt, the chairman, is now able to announce the amalgamation of his hospital with the Liverpool Children's Infirmary, from which each is likely to benefit. It may lead to the fulfilment of the plan for 500 beds, and his expense could be met if only it were possible, here as elsewhere, to increase the range of hospital supporters. In general these are generous but few, and it is easier to draw on their generosity than to widen their number. Dr. Macalister, one of the founders, records that the first patient, who was received as an incurable in 1890, is now the healthy mother of a healthy child. The percentage of cures of all cases is now, he said, seventy, and he pleaded for 500 beds and the establishment of a teaching school for children. In regard to the amalgamation, Dr. Macalister regarded it as an earnest of general unification.

### WHO WILL GET THE £400,000?

The treasurers and secretaries of the London hospitals are eagerly looking forward to more information as to the will of the late Mr. William Shepherd, of Clapham Park, and that gentleman's executors are at present much sought-after people. Mr. Shepherd, who was for many years chairman of the Bermondsey Vestry and Board of Guardians and some time president of the London Master Builders Association, left property to the value of £600,316, the net personality being £481,934. After some comparatively small legacies have been paid, the residue of the property is to be devoted to such hospitals in London as the testator may direct or the executors select. The wording of the will makes it possible that Mr. Shepherd has left written instructions on the subject, but, whatever is the case, it is clear that some London hospitals are in for a good windfall.

### ANOTHER HOSPITAL OF RECOVERY.

Summerlee, a large house at East Finchley, which has, throughout the war, been used by the Red Cross Society as an auxiliary hospital, is by the generosity of Mr. Kennedy Jones to be retained in the service of the nation, at least for the next two years, as a home of recovery for discharged sailors and soldiers and their families. This work is to be carried out by the Great Northern Central Hospital, one of the metropolitan general hospitals without a medical school which has been much in the public eye recently. At a recent meeting the new hospital was taken over by the Marquis of Northampton, the chairman of the Great Northern Central, who thanked Mr. Jones and the committee of the auxiliary hospital who are lending the equipment and who had also given £1,000, the balance of their funds. To this sum was added a gift of £500 from Mr. Kennedy Jones, who also promised to add his Parliamentary salary of £400. The Islington section of the Union of London Retail Meat Trades have presented their War Motor Ambulance to the Great Northern Central Hospital and the drapers of the neighbourhood have already subscribed £800 to the War Memorial Extension.

### AMALGAMATION REJECTED AT PORTSMOUTH.

In these days, when the amalgamation of hospitals is not unusual, it is interesting to record the reasons which have led the Portsmouth Ear and Eye Hospital to reject the proposal of the Royal Hospital for amalgamation. Mr. H. Dummer, the Chairman of the Eye Hospital, said that the investigation of himself and his colleagues showed that the Royal could give the Eye Hospital no additional accommodation, and that if the Royal came to the Eye Hospital at least four wards and a new theatre would be required. He did not see how amalgamation would reduce their expenses, nor by its means, he said, could the staff be curtailed. The Eye, unlike the Royal, Hospital was a county institution, whose patients came from outlying districts, and he feared that their donations would be lost if amalgamation took place. Each proposal of this kind must be judged on its own merits, and only very careful investigation can decide the point. But, without criticising Mr. Dummer's view in general, we are surprised to hear him say that amalgamation would not reduce the staff. It is true that the members of one staff might be preferred to those of another; but does he really mean to suggest that, if amalgamation had been decided on, the staffs of both institutions would still be required?

### THE POSITION AT NORWICH.

The process of returning to pre-war conditions is proving difficult for the Norfolk and Norwich Hospital. The withdrawal of the grants received for the former military patients diminishes the receipts by £15,000 a year. The extra wards built for them are largely still in use for discharged soldiers. At present the private nursing staff is still unrevived, because there has been nowhere to house its members. To remedy this, Surrey House, in Newmarket Road, has been bought, and will be used for private nurses when the necessary alterations have been made. It is hoped, if the latest appeal is successful, to extend the present hospital and to form an orthopædic department. Meantime, the waiting-list continues to grow, but Dr. S. Barton has declared that no urgent cases are kept waiting, and if this is really so, the Norfolk and Norwich Hospital is more fortunately placed than other voluntary hospitals at present.

### THE ORGANISATION OF SUMMER FETES.

We regret occasionally to see that hospital fêtes in the country sometimes attract only a small attendance, notwithstanding the pains taken by willing workers to make them attractive. It is not easy to remedy this in the remote country, where it is difficult to make local events known, but in districts large enough to possess daily local newspapers the remedy is simple. A superintendent, who has been very successful in appeals of all kinds, tells us that several hundreds have been added to the attendance at fêtes held on behalf of his hospital, by the appearance in the local papers of a paragraph on a number of successive days. It is the old story of a "cumulative impression." Writers are apt to tire before the public of the same; indeed, it might be said that until the public has been reduced to the level of the writer the subject has not been impressed upon it. These paragraphs should appear in the general body of the paper as part of the general news. But they should appear regularly, if possible on a number of successive days.

### TUBERCULOUS SERVICE MEN AT MARGATE.

The new wing in connection with the Royal Sea-Bathing Hospital, Margate, which was begun in consequence of the request of the Local Government Board that the hospital should treat men discharged from both services with surgical tuberculosis, is nearing completion. It should be ready for use in the autumn, and will accommodate 68 patients and the additional nurses required. In addition to the grant from the Local Government Board, a sum of £13,000 is asked for. Since this will be, perhaps, the first hospital building to be finished after the war, there should be a local pride in opening it free from debt.

### A COMPLETED WAR MEMORIAL.

Bognor can be said to be one of the first to have completed its War Memorial, which already is in active use. The Cottage Hospital was opened on July 16, being dedicated "in the names of all those brave men who have fallen in the war, who had given their lives that we might still retain our liberty." But this signal honour in so quickly commemorating the war is due mainly to Mr. James Fleming, who gave £7,000 towards the scheme. Assistance has been forthcoming in furnishing the hospital, but much is still needed. In view, however, of Mr. Fleming's munificence, there should be a ready response to the hint which that gentleman threw out at the opening ceremony that he would much like to see a laboratory, an *x*-ray installation, and a convalescent ward as part and parcel of the hospital.

### TUNSTALL'S COTTAGE HOSPITAL.

The committee in charge of the Potteries War Memorial Scheme has accepted the request of the Tunstall committee for the inclusion of a cottage hospital there. A sum of £5,000 has been earmarked for this purpose. The plan is interesting because Tunstall is at the north end of the borough, and therefore furthest from the North Staffordshire Infirmary. The local wish for a cottage hospital is strong and of long standing, and consequently there is every prospect that the central committee will receive generous support from Tunstall now that a cottage hospital is to be built there.

### THE CULTIVATION OF LEGACIES.

The need for our column headed "The Cultivation of Legacies" could not be better illustrated than by the recent financial history of Queen Charlotte's Hospital. This institution, perhaps the most famous of all lying-in hospitals, yet confesses a sensational fall in income derived from legacies. For the past five years, only £323 has been received from this source, although during the previous ten years the annual yield from legacies was over £2,500. In other words, the overdraft of £10,000 at the bank would not have existed had legacies not dropped, but merely maintained their average yield. The need to cultivate legacies is, therefore, obvious. Apart from the art of treating every visitor, every correspondent as a possible testator in favour of the hospital, solicitors and testators in general have to be kept in mind. These people with money to bequeath or advice to give to intending testators to charity, consult, when in doubt, certain established sources of information. The more precisely interested refer to the statistical information and record of work contained in "Burdett's Hospitals and Charities," and turn, among other places, to the page devoted to this subject in *The Hospital*. Its advantage is that it is always there, that it forms a pile of information, and that the information given is as concise as a secretary knows how to make it. Really, the fall in legacies is preventable. Legacies fluctuate, but they will maintain an average provided the sources from which they come are continually nursed.

### A CROYDON SURGEON'S BEQUESTS.

By the will of the late Dr. Henry John Strong, J.P., of Worthing, formerly a surgeon in Croydon, a number of valuable legacies are left to various institutions and charities. Subject to the payment of a few legacies, the testator left the residue of his estate, estimated at a total value of £47,750, to be divided into twenty equal parts. Of these twenty shares the Royal Medical Benevolent College, Epsom, is to have six; the Whitgift Almshouses and Pension Foundation (Croydon) takes three; while two shares each are allotted to the Croydon General Hospital and to the technical education of soldiers and sailors wholly or partially blinded in the War. Of the remaining shares, one each goes to the Whitgift Educational Foundation, the Worthing Hospital, the National Benevolent Institution, the Invalid Children's Aid Association, and the Royal Masonic Benevolent Institution.

### MARRIAGE OF SANATORIA SUPERINTENDENTS

A PROTEST has been made against the stipulation that candidates for the post of medical superintendent at Middleton-in-Wharfedale, under the West Riding County Council must be unmarried. This post, which was lately advertised, is one of several limited to single men, and this explains the feeling which has been excited. The sanatorium authorities, that is, the West Riding County Council, have informed the *Yorkshire Post*, where the protest appeared, that the only reason for the stipulation is the absence of accommodation for married doctors. Since, however, the "administration"—which presumably means the administrative—blocks is still unfinished, there seems no reason why married men should not be accepted as candidates so long as the limited conditions of the quarters at present available is explained to them. Men really equipped for such posts by special study and clinical experience are still few, indeed technical qualifications are too little insisted on; there is all the more reason, therefore, to exclude no candidate on artificial grounds. The County Council should hurry up and complete the administrative block. It would even be patriotic, as all building is at present.

### CONVALESCENT HOMES AND SECTARIANISM.

ONE of the last acts of the Local Government Board before it was merged in the Ministry of Health, was to decline the request of the Southwark Board of Guardians to sanction a convalescent home for their patients. The Board pointed out that in a number of instances convalescent homes and institutions were charities, and not conducted on a profit-making basis. Unfortunately these are few, and there is considerable difficulty in the Guardians getting admissions to them. There is also a further drawback that these are often sectarian. Dealing with people of all kinds and creeds, the Guardians felt that if they had their own convalescent home, not only would they save a considerable sum of money, but also rid themselves of some petty annoyances to which they and their patients have been subjected. We give the Guardians' point of view because hospitals also receive patients of all kinds and creeds, and yet very rarely experience sectarian trouble. Why, then, are those who manage convalescent homes more difficult to deal with, and why is the convalescent patient especially "annoyed"?

### AMERICAN SURGEONS IN FRANCE.

MUCH has been heard of the whole-hearted co-operation of British and American medical service during the later years of the war, but there is sometimes risk of forgetting that much earlier we received considerable assistance in medical matters from our Allies. Sir Anthony Bowlby draws attention to the fact in a recent acknowledgment in the *Times*, and reminds us that an American unit from Harvard University completely staffed one of our general hospitals in France as early as 1915, and in 1917 six more British hospitals in France were staffed by the United States. Also, that at about the same time the B.E.F. had from one to two hundred American surgeons working with regiments, field ambulances, and casualty clearing stations, and that not a few of these men were either killed or wounded while serving under the British flag.

### A LOSS TO WALTHAMSTOW HOSPITAL.

WALTHAMSTOW HOSPITAL sustains another loss by the death of Dr. Frank Collins, who for many years has been one of the staff. Dr. Collins acted as the hospital's Hon. Medical Officer for Wanstead, and from the time of his first connection with the hospital showed the keenest interest in it. He was also, and until recently, for many years Deputy Coroner for the district.

### DR. TOOGOOD'S RESIGNATION.

The retirement of Dr. Frederick Sherman Toogood, O.B.E., after twenty-five years' service as medical superintendent of Lewisham Infirmary, was marked by a dinner in his honour and the presentation of a gold watch and an oak bookcase. The clerk to the Guardians, Mr. W. R. Owen, took the chair, and said that it was to Dr. Toogood that Lewisham Infirmary owes its high position. In reply Dr. Toogood said that he had been for thirty-three years in the Poor-Law Service. It was at that time as to its nursing deplorable, and its medical service was scanty. Personally he started with the ambition that the Poor-Law infirmary should be as well equipped and staffed as any hospital in London, and he thinks that Lewisham Infirmary has reached that standard. Dr. Toogood's successor is Dr. Humphrey Nockolds, D.S.O.

### THE LATE LORD CADOGAN.

The Chelsea Hospital for Women has now received a portrait of the late Earl Cadogan, its valued friend for many years. It was presented by the Marquess of Londonderry, the president, who recalled that it was Lord Cadogan who laid the foundation of the new hospital where they were assembled, and that the site was one of his many munificent gifts to the institution. The portrait, which is set in a panel over the board-room mantelpiece, is a copy by the Marchese Nerli of Mr. Solomon J. Solomon's picture.

### THE NEW SECRETARY OF GLOUCESTER ROYAL INFIRMARY.

The post of Secretary to the Gloucester Royal Infirmary is for the moment unoccupied though filled. After the resignation of Mr. Pike last January, the vacancy was advertised, and the general committee appointed Capt. Gladstone Hurford, of Knowle, Bristol, at a salary of £250 a year. He is still awaiting demobilisation.

### THE SALARIES OF LAUNDRYMAIDS.

The committee of Swansea Hospital has decided to increase the salaries of laundrymaids and of all the domestic staff who live in by 25 per cent. The laundry is only one of the domestic problems with which homes, whether public or private, are confronted, and the commercial laundries are mostly so short of staff that they decline new customers. We have always maintained that the improvement of the conditions of nursing would be followed by a similar improvement in the prospects of the domestic staff, and the war has hastened both improvements considerably.

### WOMEN STUDENTS AND THE LONDON.

In addressing the students at the meeting of the College Board of the London Hospital, Mr. Marlborough Pryor stated that the year 1918-19 was as momentous in the history of the hospital as in the great world outside. During this year arrangements had been completed for the teaching of the purely scientific subjects at East London College, a chair of bacteriology had been endowed by the Goldsmiths' Company, and finally women students had been admitted on an equal footing to that of men. He stated that it was specially gratifying to him to have been able to present no less than four prizes and five honorary certificates to women students. He made a special appeal to women students to consider the claims and attractions of research, for he considered that in many ways, by their delicate sense of touch, their quick perceptive and intuitive powers, they had an advantage over men. He also believed that they were more self-denying and more ready, therefore, to devote themselves to such work. He trusted that funds would be available from national or other sources to enable such research to be prosecuted.

### EDUCATION IN HOSPITAL.

It is now nearly six months since an Education Officer was appointed to the Central (Military) Hospital, Old Town, Eastbourne. Since then the work, which began to lessen the *ennui* of military life, has much extended, and now, apart from the trades, such subjects as Greek, commercial Spanish, English, arithmetic, and landscape gardening are being taught. Two-thirds of the men in hospital attend one or other of the classes, which seem to be as popular now as they were suspect at first. This is typical of what is going on and should prove to many men the most valuable of their military experiences.

### HOW TO DEAL WITH PATIENTS' LEAVINGS.

The answer to the question: What happens to patients' leavings? is still incomplete. Hospitals which keep pigs have no difficulty in disposing of these scraps of food. But what about those in towns which have no piggeries? A secretary says that the scraps can be sold to pig owners. Is there not an alternative?

### THIS WEEK'S DRUG MARKET.

It goes without saying that in consequence of the intervention of the Peace holiday business has been unsettled since the last report appeared. Notwithstanding the interruption, however, there is already a good deal of inquiry; in fact, business has been resumed with little delay. Generally speaking, the tendency of prices is still in an upward direction, but it is unusually difficult to express anything like a useful opinion as to what the immediate future holds in store so far as the drug market is concerned, as there are so many uncertain factors. In the meantime, prices undoubtedly have a firmer tone. Menthol is substantially dearer, and the same applies to Japanese peppermint oil. Cloves continue to move upwards in price, and clove oil is likely to follow suit. There has been another rise in the price of quicksilver, and this has again been followed by higher quotations for the salts of mercury. Other articles that are dearer include vanillin, castor oil, glucose, milk, sugar, podophyllum root, glucose, salicylic acid, and aspirin. One of the few articles that have a downward tendency is opium, but so far there has been no notable reduction in the alkaloids of opium.

